# Multi-omics Comparative Analysis of *Streptomyces* Mutants Obtained by Iterative Atmosphere and Room-Temperature Plasma Mutagenesis

**DOI:** 10.3389/fmicb.2020.630309

**Published:** 2021-01-28

**Authors:** Tan Liu, Zhiyong Huang, Xi Gui, Wei Xiang, Yubo Jin, Jun Chen, Jing Zhao

**Affiliations:** ^1^College of Ocean and Earth Science, Xiamen University, Xiamen, China; ^2^Tianjin Institute of Industrial Biotechnology, Chinese Academy of Sciences, Tianjin, China; ^3^Fujian Collaborative Innovation Center for Exploitation and Utilization of Marine Biological Resources, Xiamen, China

**Keywords:** ARTP mutagenesis, awaken cryptic gene clusters, antibacterial activity, transcriptome comparative analysis, metabolome comparative analysis

## Abstract

Sponges, the most primitive multicellular animals, contain a large number of unique microbial communities. Sponge-associated microorganisms, particularly actinomyces, have the potential to produce diverse active natural products. However, a large number of silent secondary metabolic gene clusters have failed to be revived under laboratory culture conditions. In this study, iterative atmospheric room-temperature plasma. (ARTP) mutagenesis coupled with multi-omics conjoint analysis was adopted to activate the inactive wild *Streptomyces* strain. The desirable exposure time employed in this study was 75 s to obtain the appropriate lethality rate (94%) and mutation positive rate (40.94%). After three iterations of ARTP mutagenesis, the proportion of mutants exhibiting antibacterial activities significantly increased by 75%. Transcriptome analysis further demonstrated that the differential gene expression levels of encoding type I lasso peptide aborycin had a significant upward trend in active mutants compared with wild-type strains, which was confirmed by LC-MS results with a relative molecular mass of 1082.43 ([M + 2H]^2+^ at *m/z* = 2164.86). Moreover, metabolome comparative analysis of the mutant and wild-type strains showed that four spectra or mass peaks presented obvious differences in terms of the total ion count or extracting ion current profiles with each peak corresponding to a specific compound exhibiting moderate antibacterial activity against Gram-positive indicators. Taken together, our data suggest that the ARTP treatment method coupled with multi-omics profiling analysis could be used to estimate the valid active molecules of metabolites from microbial crudes without requiring a time-consuming isolation process.

## Introduction

Marine sponges (phylum Porifera) are among the oldest Metazoans on Earth (Webster and Taylor, 2012). Due to their marine benthic environment and highly efficient filter-feeding abilities, sponges harbor abundant microorganisms, including bacteria, fungi, archaea, and microalgae (Cheng et al., 2020). In fact, our previous studies identified 363 strains from 49 sponge species, including 92 actinobacterial isolates with diverse antibacterial potential, in which most streptomycetes displayed moderate to strong antibacterial activity by using the minimal medium that inhibited several Gram-positive and -negative indicators (Liu et al., 2019). These marine symbionts can account for up to 40–60% of the sponges’ total volume, most of which contribute to the nutrients and survival of the host (Hentschel et al., 2012). Moreover, many sponge-derived bioactive substances are synthesized, or partly synthesized, by sponge-associated microbial organisms rather than the sponges (Thomas et al., 2010). To date, more than 15,000 natural compounds have been obtained from marine sponges and symbionts, including alkaloids, peptides, terpenes, and polyketides (PKs) (Mehbub et al., 2014). Among these marine microorganisms, actinomycetes are the largest producers of secondary metabolites, with more than 10,000 compounds (approximately 45%) (Genilloud, 2017). Indeed, actinomycetes-associated secondary metabolites possess antimicrobial activity, 75% of which were isolated from *Streptomyces* (Avignone-Rossa et al., 2013).

Although many natural compounds have been discovered, they do not represent the full richness of what the marine actinobacterial groups can provide (Gerwick and Moore, 2012). However, isolating and characterizing new compounds with novel structures or activities has become increasing challenging. In recent decades, only two top-ranking microbial natural antibiotics, retapamulin (from fungal terpenoid pleuromutilin) and fidaxomicin (tiacumicin from *Actinoplanes deccanensis* tiacumicin), which only act against Gram-positive bacteria, have been launched (Mullane and Gorbach, 2011; Sun et al., 2018). Most other compounds are either derived from known antibiotics or are unable to meet the required standards of clinical trials. One associated drawback is that more than 99% of bacteria cannot be cultivated under laboratory conditions (Vartoukian et al., 2010). Additionally, secondary metabolite gene clusters generally remain silent or cryptic when strains are cultivated in the laboratory, likely due to a lack of environmental pressures (Seyedsayamdost, 2018). In fact, recent advances in whole genome sequencing and bioinformatics have revealed an abundance of cryptic biosynthetic gene clusters (BGCs), as much as 25–70 within actinobacterial genomes (Lee et al., 2020b). Therefore, it is crucial to awaken poorly expressed or silent gene clusters to effectively exploit the biosynthetic potential of these strains for the production of new compounds.

Unlike certain diverse molecular strategies capable of awakening these silent gene clusters, such as ribosome engineering, manipulation of regulatory genes, overexpression or replacement of promoters, and epigenetic perturbation, random gene mutations remain the only feasible method when the genomic sites of the desired phenotype are unknown, or the genetic regulation is complex (Rutledge and Challis, 2015). Unlike traditional mutation breeding methods, such as physical mutation, chemical mutation, DNA recombination, and protoplast fusion, atmospheric room-temperature plasma (ARTP) is a platform for microbial mutagenesis based on atmospheric-pressure discharge plasma with higher mutation rates and low treatment temperatures (Zhang et al., 2014). As of 2018, at least 14 fungi, and 24 bacteria, plants, and other microbial communities were successfully altered to improve the tolerance of their microorganisms to certain growth conditions, increasing biomass and optimizing relevant parameters or enhancing the production of enzyme activity or medicinal chemicals (Ottenheim et al., 2018). The ARTP system causes greater damage to DNA that results in various structural changes to the oligonucleotides, which accounts for the higher mutation rate compared to other methods, such as UV radiation or chemical mutagens (Wang et al., 2020).

Moreover, ongoing advances in gene mining, comparative transcriptomics, metabolomics, and other bioinformatic tools have made them promising methods for discovering natural products from actinomycetes (Boddy, 2014). By combining genome mining approaches with antiSMASH tools, bacterial PKs and non-ribosomal peptides (NRPs), as well as complex secondary metabolites with antimicrobial, antiviral, anti-infective, or anticancer properties, have been identified (Lee et al., 2020a). These new characterization methods have also predicted new biosynthetic pathways and their related natural compounds. For instance, Streptoseomycin and its BGC were identified from marine prawn symbiotic actinomycetes by genome mining (Zhang et al., 2018). Moreover, the metagenomic sequencing of sponge *Mycale hentscheli* symbionts identified more than 100 cryptic BGCs and linked them to diverse chemical substances with anticancer or antiviral abilities (Storey et al., 2020). Additionally, some novel putative BGCs have been identified in various unculturable microorganisms. RNA-seq (transcriptome sequencing) technology has also been widely applied to compare differential expression levels of secondary metabolic gene clusters that respond to the same or different compounds with specific ecological functions under different environmental conditions. For example, comparative transcriptomics was used to predict regulon and to identify its pleiotropic regulator, like DasR, representing the “switch” between primary and secondary metabolite processes that improves yields and awakens silent pathways (Nieselt et al., 2010). Comparative transcriptome and metabolomics not only provide new methods for gene mining and contributions to revealing the biosynthetic potential of silencing secondary metabolic gene clusters, but also offer a targeted scheme for the subsequent improvement of potentially active compounds that cannot be obtained for further analysis due to the poor expression of gene clusters.

In this study, iterative ARTP mutagenesis was applied to sponge-derived *Streptomyces* sp. MG 010 to obtain mutants with increased antimicrobial activity. Based on the gene mining, transcriptome, and metabolomic results, the transcriptional expression levels and corresponding metabolites of the BGCs encoding antimicrobial products were comparatively analyzed between mutants and the wild-type strain. This may provide valuable guidance for further studies on activating the silent or cryptic gene clusters of actinobacteria, while supporting further research on the related mechanisms of compound biosynthesis and the targeted development of bioactive products.

## Materials and Methods

### Cultivation of *Streptomyces* sp. Strains

*Streptomyces* sp. MG010 was isolated from the marine sponge *Mycale* sp. located off the coast of Fujian, China. Gram-positive (*Staphylococcus aureus* ATCC 6538 and *Bacillus subtilis* ATCC 6633) and Gram-negative bacteria (*Escherichia coli* ATCC 25922 and *Vibrio alginolyticus* ATCC 33783) were used as indicators for the antibacterial activity assays.

ISP2 medium and LB medium were used for strain screening, fermentation, and antimicrobial bioassays. All media were sterilized at 115°C for 30 min before use (Bertani, 1951; Shirling and Gottlieb, 1966).

### Preparation of Single Spore Suspension

*Streptomyces* sp. MG010 strain was inoculated into ISP2 solid medium and incubated at 28°C for 10 days. The mature spores were harvested using sterile cotton swabs scraped over the medium surface, and then washed twice with sterile saline. The spore pellets were then transferred to an Eppendorf tube with glass beads, and the spore chains were disrupted by shaking for 10 min. After centrifuging at 12,000 × *g* for 10 min and discarding the supernatant, the spore precipitate was carefully resuspended in 1 mL of sterile saline. The monospore suspension was counted using a blood counting chamber and the concentration was adjusted to 10^7^–10^8^ CFU/mL.

### ARTP Mutagenesis

The operating parameters were as follows: an input voltage of 110 W, distance of 2 mm between the plasma torch nozzle exit and sample plate, pure helium used as the working gas, a gas flow rate of 10 slpm, and the temperature of the plasma jet was below 40°C. The monospore suspension (10 μL) was poured into a sterilized stainless-steel plate (6 mm diameter) and exposed to the ARTP plasma jet (SiQingYuan Inc., Wuxi, China) for different treatment times: 0, 30, 45, 60, 75, 90, 105, 120, 150, and 180 s.

To obtain the optimum mutation, the lethality and positivity rates of spores under different mutant treatment times were determined according to the following equations:

(1) Lethalityrate(%)=(A-B)/A×100%

where A is the total colony count of the sample without treatment and B represents the total colony count after the different mutant treatment times with ARTP.

(2) Positiverate(%)=C/D×100%

where C is the total colony count of the mutants with increased antibacterial activity and D represents the total colony count of mutants with varying antibacterial activity.

### Screening Process of the Iterative Mutant Strains

**FIGURE 1:**
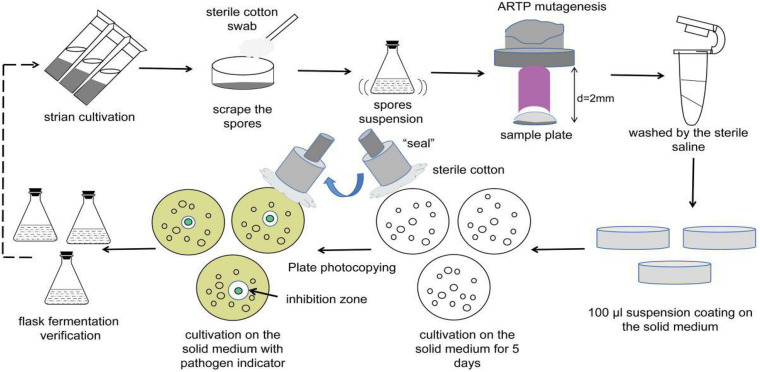
Flow diagram of ARTP mutagenesis.

After treatment, the steel plates were placed into new 1.5 mL Eppendorf tubes and washed with 1 mL sterile saline. Then, 100 μL spore suspension was separately transferred onto ISP2 solid medium and cultured at 28°C for 5 days. Pure mutant colonies from the solid medium were selected and inoculated onto LB solid medium with typical indicative bacteria at 37°C for 1 day using the replica plating method. The diameters of the inhibitory zones were observed and measured. Next, 100 μL of the cell-free supernatants of the fermentation broths from potentially active mutants was drawn out for verification of the antibacterial activity using the Oxford Cup assay. The top mutants with increased antimicrobial activity were selected as the original isolates for the next round of ARTP mutagenesis. A total of three mutagenesis iterations were performed along with the screening methods (Figure 1). In addition, the genetic stability of the identified mutant isolates was evaluated by eight rounds of serial sub-cultivations.

### RNA Extraction, and Transcriptome Sequencing and Analysis

The 16S rRNA gene sequences (∼1500 bp) of the MG010 strain were amplified and analyzed by the National Center of Biotechnology Information (NCBI) website^1^ to select the most related reference strains. The nucleotide sequence data showed that *Streptomyces* sp. MG010 (GenBank accession number: MW186238) in this study shared 99% homology with *Streptomyces variabilis* strain HNS054 (GenBank accession number: ASM104418v1). Therefore, the *S. variabilis* strain HNS054 was chosen as the reference genome for subsequent experiments. Antibiotics and secondary metabolite analysis shell (antiSMASH) version 3.0.5 was used to analyze the potential to produce secondary metabolites.

Total RNA was extracted and purified from the wild-type strains (labeled NA1, NA2, and NA3) without testing their bioactivity against indicative bacteria, weakly bioactive mutants (labeled I1, I2, and I3), or the strongest bioactive mutants (labeled M1, M2, and M3) in the three parallel samples using the TIANGEN RNAprep Pure Micro Kit (TianGen, China) and RNA purification kit (TianGen, China), according to the manufacturer’s instructions. The purity and concentration of the RNA (rRNA 28s/18s) were evaluated using a NanoDrop 2000 spectrophotometer (NanoDrop, Thermo Scientific, United States), and the integrity of the RNA was assessed using agarose gel electrophoresis. Then, all the RNA samples were subjected to RNA-seq library preparation and directed sequencing on the Illumina HiSeq platform (Meiji, China).

After filtering the low-quality sequences, a high content of unidentified nucleotides N, and adaptor-polluted sequences, clean reads were assembled using the software provided by sequencing platform Illumina. Clean reads were mapped to the reference *Streptomyces* sp. and the HNS054 genome (accession number: NZ_LDZX00000000.1, available at: https://www.ncbi.nlm.nih.gov/nuccore/NZ_LDZX00000000.1) using the software Bowtie2^2^. The abundance of gene transcripts from RNA-seq was quantified using the software RSEM package^3^. RPKM (reads per kilobase of exon model per million mapped reads) was used to indicate the abundance of a transcript. The edge R^4^ was used to analyze differential expression analysis (fold change) and the related significance level between the wild and mutant strains. The false discovery rate (FDR) values below 0.05 or log_2_ | fold change| ≥ 1 were considered to be significantly differentially expressed transcripts.

Gene functions were annotated by online programs and databases, including Gene Ontology (GO), Clusters of Orthologous Groups of protein (COG), and KEGG. Among these, GO terms enriched differentially expressed genes (DEGs) were analyzed using the Blast2GO software package^5^. The GO terms with significant expression (*p*-value < 0.05) were also enriched in KEGG pathways^6^; COG^7^ terms were used as tools for functional annotation, classification, and protein evolution analysis. Other databases such as Nt (NCBI non-redundant nucleotide sequences), Nr (NCBI non-redundant protein sequences), and Pfam (Protein family) were also used to annotate gene functions.

### Quantitative Real-Time (qRT)-PCR Analysis

A total of nine RNA samples were collected for quantitative real-time (qRT)-PCR analysis to verify the transcriptome data. The primers used for qRT-PCR were designed using the Premier 5 software. The detailed information for the primers is listed in Supplementary Table S3. The qRT-PCR reaction system was performed following the manufacturer’s instructions using the SYBR Premix EX Tag kit (Takara, Japan), containing 10 μL of 2 × SYBR Premix EX Tag, 2 μL of each cDNA sample, 0.5 μL of Primer (10 μM), and added RNase Free H_2_O up to a final volume of 20 μL. The reaction conditions were as follows: 95°C for 2 min; 40 cycles (95°C for 5 s; 60°C for 30 s) and 72°C for 30 s. The melting curve conditions were as follows: 95°C for 15 s; 60°C for 60 s; and 95°C for 15 s. The 16S rRNA of the wild strain was used as the reference gene in this study. QuantStudio 6 (Thermo Fisher Scientific, United States) was used to carry out all qRT-PCR reactions by detecting the SYBR fluorescence signal strength. Primer Quest Tool software was used to design primers with a product length of 100–300 bp (Invitrogen, ShangHai). The 2^−ΔΔ*Ct*^ method was used for data processing.

### LC-MS Analyses of Wild Strain and Mutants

The wild-type strain (NA), weakly mutant strains (IA), and maximum antimicrobial activity of the mutant strains (MA) were first grown on ISP2 medium at 28°C for 5 days, after which the spores were inoculated into a 2.5 L Erlenmeyer flask containing 2.5 L ISP2 medium at 220 rpm and 28°C for 7 days. The crude extracts were obtained by extracting the fermentation broths with equal volumes of ethyl acetate and then butanone three times. The clarified supernatant was obtained by centrifuging the concentrates at 12,000 × *g* for 5 min. Both the ethyl acetate and butanone layers were collected and evaporated on a rotatory evaporator, and the concentrates were resolved in methanol (500 μL). The samples were adjusted to 5 mg/mL and analyzed by LC-MS with a linear gradient of 5–95% solvent B (solvent B: 0.1% formic acid + 100% CH_3_CN; solvent A: 0.1% formic acid in H_2_O) over 40 min at a rate of 1 mL/min with a UV length of 254 nm.

The free software MZmine2^8^ was used to assess chromatograms by detecting, deconvoluting, aligning, and normalizing the data. Only those peaks with a high-quality mass intensity were retained, while the rest were discarded.

### Antibacterial Activity Assay

The major predicted active fractions were collected by semi-preparative HPLC with a C_18_ column using a CH_3_CN–H_2_O elution system as previously described to identify the relatively pure peaks. The fractions were then dissolved in DMSO and adjusted to a uniform concentration of 100 μg/mL. Bacterial indicators were selected for antibacterial activity assays using the disk paper method. After cultivation for 24 h, the diameters of the bacteriostatic circles were measured.

## Results

### Identified Mutations and Screening of the Mutants

**FIGURE 2:**
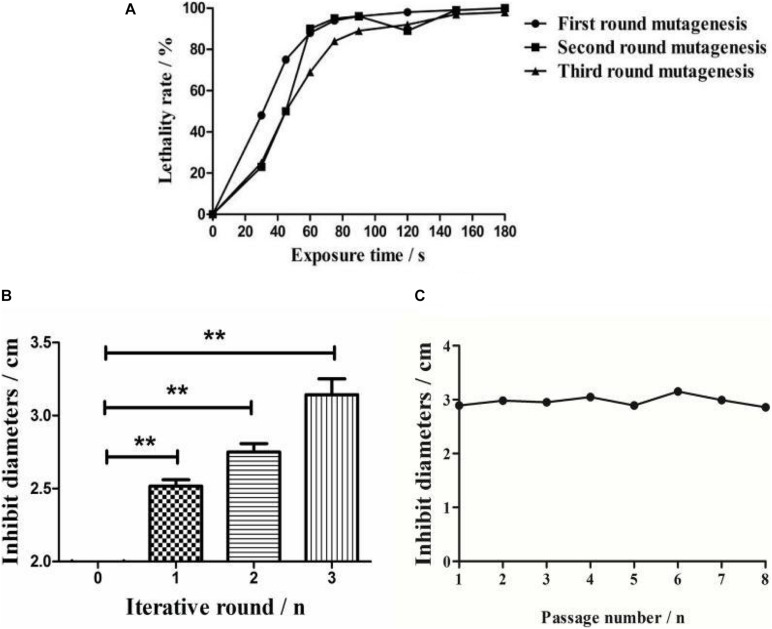
Comparative analysis of ARTP mutagenesis process. **(A)** The lethality rate of the wild-type strain under different ARTP actuation durations. **(B)** Bacterial inhibition diameters of the wild-type strain and the active mutant strains after three iterations of ARTP mutagenesis. **P < 0.01. **(C)** Generational fermentation of the mutant strains.

The survival rate of *Streptomyces* sp. decreased with an increase in the mutagenesis time (Figure 2A). The lethal rates sharply increased when strains were exposed to ARTP before 75 s, and were observed to plateau at a treatment time of 75 s, with the death rate reaching 94% for the protection of the spore cell wall. Meanwhile, no spores survived treatments of 90 s or longer. Therefore, 75 s was chosen as the optimal exposure time for all subsequent experiments.

After three mutagenesis iterations, a total of 813 mutants were selected for antimicrobial activity screening. The mutation rates decreased as the positive mutation rates increased from 8.97 to 40.94%, indicating that interactive mutagenesis increased the possibility of emerging active strains (Supplementary Table S1). The antibacterial activities were then compared among the optimal mutants in each round (Figure 2B). Compared with the wild-type strain, the inhibitory diameter increased by 68% (first round), 71% (second round), and 75% (third round) after each round of ARTP treatment under the same culture conditions.

### Mutant Stability

To detect the genetic stability of the mutant strains, eight rounds of subculture shake flask fermentation of the optimal mutants were conducted. The results indicated that the inhibitory activity of the identified mutant strains retained effective genetic stability (Figure 2C).

Partial characteristics, including the thermal stability and protease degradation activity, of one selected representative mutant were analyzed. The specific experimental methods are listed in Supplementary Figure S5. The bioactivity of the fermentation broth against indicative bacteria did not significantly differ when placed at 80°C for 4 h; however, no activity was observed above 80°C (Supplementary Figures S5A,B). The active substances from the fermentation broth were not sensitive to trypsin or chymotrypsin (Supplementary Figure S5C). Briefly, the results showed that the active substances of the mutant strains in the fermentation broth had excellent thermal stability and resistance to protease degradation.

### RNA Sequencing and Comparative Analyses of Transcriptomic Profiles

The transcriptome of nine mutants (NA, IA, and MA in three parallels, respectively) were sequenced using the RNA-seq Illumina platform. In total, 177,898,864 clean reads were obtained from the NA, IA, and MA groups, among which 164,556,861 reads had a mapped ratio larger than 80%. The correlation coefficient between the wild strain and the mutants showed good quality of the collected cDNA reads (Supplementary Figure S1A). Compared with the wild strain, the clean reads mapping to the unique genes demonstrated that 1712 transcript genes of the IA mutants were significantly changed, including 956 upregulated and 756 downregulated genes, while 1812 transcript genes of the MA mutants were significantly changed, including 1145 upregulated and 676 downregulated genes (Supplementary Figure S1B). The significance of these gene transcriptions was determined by both FDR < 0.05 and | log_2_FC| ≥ 1.

Seven of the total 825 unigenes were annotated to three major functional groups using the GO categories, including biological process (4584 unigenes), molecular function (1900 unigenes), and cellular component (1341 unigenes; Supplementary Figure S2A). Comparing the NA and MA mutant strains, the most DEGs were found to be associated with biological processes, with equal numbers of upregulated and downregulated genes. Between the IA and MA mutants, remarkable changes were observed, with more upregulated than downregulated genes (Supplementary Table S2). Based on the DEGs, five of the total 123 unigenes were assigned to 23 function units based on the COG function annotation, with the largest number assigned to transcription (387 unigenes), accounting for 7.55% (Supplementary Figure S2B). A total of 5051 unigenes were also annotated to KEGG pathways involved in metabolic pathways (815 unigenes) and biosynthesis of secondary metabolites (370 unigenes), among which 20 pathways were significantly enriched, including ABC transporters, carbon metabolism, and biosynthesis of amino acids (Supplementary Figure S2C).

### Comparison of DEGs in the Functional Gene Clusters

**FIGURE 3:**
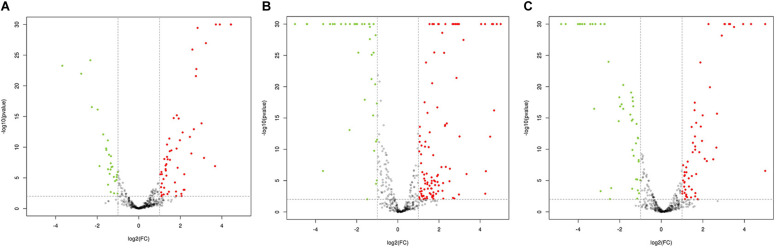
Volcano plot of pairwise comparisons between samples. **(A)** Volcano plot of the comparisons between IA (Mutants with weak activity) and MA (Mutants with maximum activity); **(B)** Volcano plot of the comparisons between NA (Wild-type strain) and MA (Mutants with maximum activity); **(C)** Volcano plot of the comparisons between NA (Wild-type strain) and IA (Mutants with weak activity).

Comparative analysis using antiSMASH version 3.0.5 of functional gene clusters between the wild-type strain and mutants revealed a total of 21 gene clusters related to secondary metabolites that were significantly differentially expressed (Table 1). Using these DEGs, we conducted pairwise comparisons (Figure 3). The transcription results responding to ARTP mutagenesis illustrated that certain metabolic pathways, such as ribosomal metabolic processes, peptide and antibiotic biosynthesis and metabolism processes, transcription factors, and protein binding transport, became more active with an increase in antibacterial activity. Several gene clusters related to PKS or NRPS compounds were also differentially expressed between the NA and MA groups, such as cluster 11 and 12 (T1 PKS-NRPS), cluster 23 (T3 PKS), cluster 37 (NRPS), and cluster 51 (T2 PKS). Additionally, type I or IIIPK synthase and peptide synthase were significantly increased, although the numeric values of these transcriptome data (FPKM) ranged from 10 (at minimum) to 100 (at maximum) on average. Cluster 42, which was predicted to produce Lantipeptide, also showed higher gene expression levels and fold change differences in ABC transporter, membrane protein, and nitroreductase family deazaflavin-dependent oxidoreductase.

**TABLE 1:** Secondary metabolite gene clusters mined from *Streptomyces* sp. MG010.

Number	Type	Location	Size (bp)
Cluster 1	Butyrolactone	3080–12,864 nt	9784
Cluster 2	Terpene	1–13,395 nt	13,394
Cluster 3	Ectoine-butyrolactone	610–16,149 nt	15,539
Cluster 5	Nrps-otherks	1–36,205 nt	36,204
Cluster 7	Lantipeptide-nrps	1–49,526 nt	49,525
Cluster 11	T1 PKS-NRPS	33,563–76,741 nt	43,178
Cluster 12	T1 PKS-NRPS	1–84,193 nt	84,192
Cluster 17	Cyclic peptide	11,685–34,178 nt	22,493
Cluster 23	T3 PKS	1–40,029 nt	40,028
Cluster 26	Ectoine	76,656–87,054 nt	10,398
Cluster 30	Terpene	62,012–86,132 nt	24,120
Cluster35	Siderophore	43,911–55,683 nt	11,772
Cluster 37	NRPS	57,631–112,729 nt	55,098
Cluster 42	Lantipeptide	232,006–254,678 nt	22,672
Cluster 51	T2 PKS	210,177–252,686 nt	42,509
Cluster 55	Bacteriocin	1–10,280 nt	10,279
Cluster 59	Phenazine	20,971–41,870 nt	20,899
Cluster 63	Terpene	79,564–100,649 nt	21,085
Cluster 69	Siderophore	256,733–269,972 nt	13,239
Cluster 72	Terpene	402,937–425,093 nt	22,156
Cluster 77	Bacteriocin	203,204–213,419 nt	10,215

**FIGURE 4:**
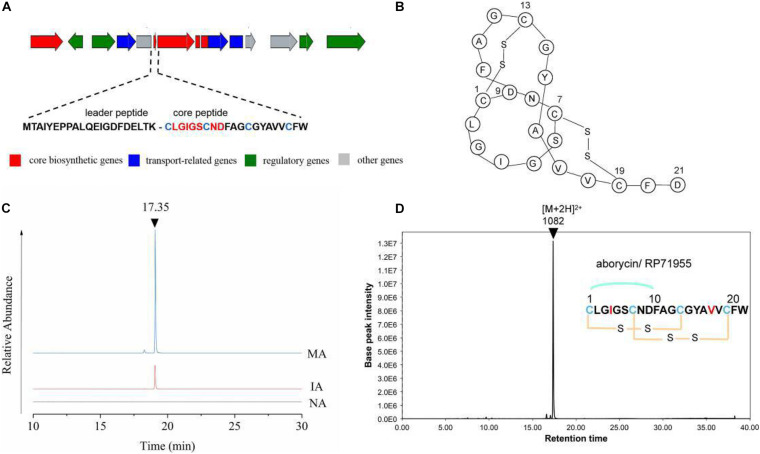
Predicated lasso peptide data. **(A)** Gene cluster structure of lasso peptide. **(B)** Secondary structure of aborycin. **(C)** Comparative HPLC analysis of the lasso peptide in the extracted crudes of NA, IA, and MA groups. **(D)** Relative molecular mass of aborycin.

The expression levels of each gene in cluster 17 showed a trend toward significant upregulation, with FPKM ranging from 10 to 3000 on average. This gene cluster, 22,493 bp in length, located in 11,658–34,178 nt, was predicted to encode and synthesize lasso peptide compounds with a molecular weight of 2164 Da using antiSMASH software (Figure 4A). The gene cluster included the DNA-binding response regulator, sensor histidine kinase, export ABC transporter ATP-binding protein, lasso RiPP family leader peptide-containing protein, lasso peptide isopeptide bond-forming cyclase, lasso peptide biosynthesis PqqD family chaperone lasso peptide biosynthesis B2 protein, and ABC transporter ATP-binding protein (Table 2). Among these, AC003_RS25355 contained the complete leader peptide and core peptide encoding the lasso peptide compounds (Figure 4B).

**TABLE 2:** Gene expression changes and function prediction of lasso peptide gene cluster.

Cluster	NA_mean_ fpkm	IA_mean_ fpkm	MA_mean_ fpkm	Predicted function
AC003_RS25335	20.08	22.39	32.52	Alpha/beta hydrolase
AC003_RS25345	173.77	144.33	339.3	DNA-binding response regulator
AC003_RS25350	79.02	87.69	68.57	Sensor histidine kinase
AC003_RS25355	15.13	233.47	3481.14	Export ABC transporter ATP-binnding protein
AC003_RS25360	11	125.51	1175.04	ABC transporter permease
AC003_RS35325	15.13	132.45	3080.16	Lasso RiPP family leader peptide-containing protein
AC003_RS25365	19.18	59.21	149.07	Lasso peptide isopeptide bond-forming cyclase
AC003_RS25370	2.59	7.7	68.71	Lasso peptide biosynthesis PqqD family chaperone
AC003_RS25375	9.07	12.59	73.23	Lasso peptide biosynthesis B2 protein
AC003_RS25380	16.16	14.33	102.59	ABC transporter ATP-binding protein
AC003_RS25385	5.51	13.73	92.72	ABC transporter
AC003_RS25390	2.99	10.56	86.5	DoxX family membrane protein
AC003_RS25400	7.75	14.07	55.72	CapA family protein
AC003_RS25405	275.92	120.82	183.81	DNA-binding response regulator
AC003_RS25415	41.58	34.11	23.66	AfsR/SARP family transcriptional regulator

### qRT-PCR Results

To validate the confidence of the RNA-seq data, five genes were selected randomly from the NA, IA, and MA mutant strains for qRT-PCR analysis (Supplementary Table S3). The linear regression relationship of gene expression levels was effective, with *R*^2^ of the NA, IA, and MA mutants reaching 0.9993, 0.998, and 1, respectively, while *R*^2^ of the reference 16S rRNA genes reached 0.99 (Supplementary Figure S3). The fold changes in the gene expression differences between the qRT-PCR results and transcriptomic data showed that although there was little deviation in these quantitative results, the up- and downregulated trends of these DEGs were the same (Supplementary Figure S4 and Supplementary Table S4). The other eight genes (AC003_RS25345, AC003_RS25350, AC003_RS25355, AC003_RS35325, AC003_RS25365, AC003_RS25370, AC003_RS25375, and AC003_RS25380) in cluster 17 that predicted for lasso peptide also underwent several rounds of qRT-PCR analysis. Although the low gene expression level limited these results, significant differences were observed in the gene expression fold changes between the MA and NA mutants, thereby verifying that the changes in gene expression of the lasso peptide cluster were relatively accurate.

### LC-MS and Antibacterial Activities Assay Results

The butanone-extracted samples of the wild-type strain (NA) and mutants (IA, MA) were analyzed by HPLC-MS. The results of metabolite profiling showed that the MA strain produced a higher yield of the putative lasso peptide (RT = 17.35 min) compared to the wild-type strain (NA) or IA (Figure 4C). LC-MS data further revealed a relative molecular mass of 1082.43 ([M + 2H]^2+^ at *m/z* = 2164.86) (Figure 4D). The genomics and metabolomics analyses of this signal were both targeted to the molecule aborycin (Frechet et al., 1994). Therefore, ARTP mutagenesis might awaken the silent BGC and, to some extent, improve the production of this lasso peptide.

**FIGURE 5:**
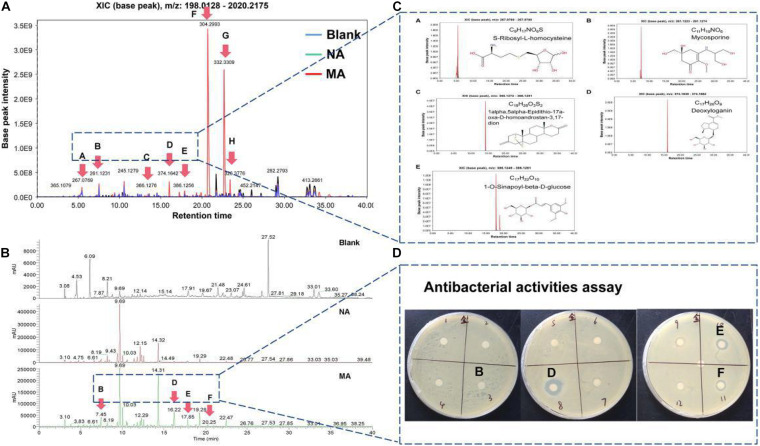
Metabolic profiles of ethyl acetate extracted crudes between the wild-type strain (NA) and mutant strain (MA). **(A)** TIC (total ion chromatogram) of the NA, MA, and blank control. **(B)** XIC (extracted ion chromatogram) of the MA. **(C)** Putative molecular formulas and structures matched to the online database. **(D)** Antibacterial activity assay results.

Additionally, the metabolic profiles of ethyl acetate extracted crudes between the wild strain (NA) and mutant strain (MA) were compared by LC-MS, determining their TIC (total ion count) and XIC (extracting ion current) (Figure 5). MA produced a series of low-molecular-weight compounds that did not contain in the NA, such as [M + H]^+^ at *m/z* = 261.1231 (RT = 7.45 min), [M + H]^+^ at *m/z* = 374.1642 (RT = 16.22 min), [M + H]^+^ at *m/z* = 386.1256 (RT = 17.85 min), and [M + H]^+^ at *m/z* = 304.2993 (RT = 20.25 min) (Figures 5A,B). To further predict these molecules, several online databases were used to identify the putative molecular formulas and structures (Figure 5C). Moreover, the spectroscopic data indicated that several unknown peaks with strong base peak intensities potentially represent new molecules with intriguing bioactivities. A panel of 12 putative fractions was used to detect potent antibacterial activities against the indicators (Figure 5D). Fractions B, D, E, and F were significantly different between the NA and MA in liquid chromatograms, among which fractions D, E, and F were against *S. aureus* with an MIC of 100 μg/mL. Meanwhile, component G and H did not exhibit antibacterial activities here, and the corresponding compounds could not be obtained from online databases, implying identification of novel compounds.

## Discussion

The first ARTP study was a report on *Streptomyces avermitilis* mutants that produced >40% more avermectins B1a than the wild-type strain (Wang et al., 2010). Over the past decade, more than 40 different microbial species involving 300 ARTP case studies have applied the ARTP mutagenesis method to improve the yield of diverse antibiotics, enzymes, and intermediate active metabolic compounds, particularly in actinomycetes (Ottenheim et al., 2018). Specifically, *Actinomyces* IN537 mutants increased acarbose yield by 62.5% compared to the original strain after treatment with ARTP (Ren et al., 2017). Moreover, iterative ARTP mutagenesis increased the transglutaminase (TGase) of bacterial fermentation by 27% with a maximum TGase activity of 5.85 U/mL (Jiang et al., 2017). Unlike most mutagenesis cases used to increase yields of known compounds for commercial high production demand, in the current study, mutants of the wild-type strain *Streptomyces* sp. MG010 were obtained by iterative ARTP with stronger antimicrobial activities, which not only indicated that silent gene clusters of *Streptomycete* strains have the potential to express active secondary metabolites, but also suggested that mutagenesis likely represented a promising method for activating gene activity corresponding to unknown compounds. Previously, *Streptomyces coelicolor* A3 was induced by rare earth elements to express nine genes related to nine BGC, although several peaks were only detected via HPLC profiling (Tanaka et al., 2010). However, these unknown active compounds, as determined in this study, have the potential to be awakened by altering a single factor to evoke a stress response and to be the most promising factor for antagonizing pathogenic bacteria (Defraine et al., 2018).

For mutagenesis, the lethality rate curve should be accurately evaluated for different species, as for actinomycetes in this study, 75 s was the optimal interval; however, for other strains, such as bacteria or fungi, this may require much shorter or longer times. The appropriate balance of the death rate and the positive rate indicated a greater possibility to obtain active mutant strains. It is advisable to ensure the appropriate initial spore concentration (10^7^–10^8^ CFU/mL) and serial dilution density to obtain single colonies on the photocopied plates. In addition, the mutant efficiency tends to increase after several rounds of mutagenesis. Unlike conventional methods, iterative mutagenesis provides a driving force for mutation by increasing the degree of damage to intracellular DNA, which could improve and maintain the rapid mutation rates (Zhang et al., 2015). Therefore, it would be much wider and faster to awaken silent gene clusters and to enhance mutation range and efficiency.

Microorganisms, particularly actinomycetes, have played an important role in the field of natural product discovery, which often harbors various cryptic BGCs that are detected under laboratory conditions by analyzing genome sequencing (Niu, 2018). Stefano et al. (2018) stated the “One strain many compounds” (OSMAC) principle in marine microorganisms that suggests that more substances could be exploited from the existing strain resources. From the perspective of natural product production, major bottlenecks remain in the discovery of novel natural compounds. Our results show that although mutants with significantly increased antibacterial activity were obtained by ARTP treatment, due to the low expression level of these secondary gene clusters, the substances were not readily identified. Thus, the differences in the LC-MS data between the wild-type strains and mutants could be in favor of identifying these possible products. The comparative analysis showed that several small molecular compounds in the metabonomics profile did not exhibit obvious changes in the transcriptomic profile. Multi-omics analysis, combining the transcriptome with metabolic regulation levels, revealed more valuable information. Lu et al. (2018) used a multi-omics integrated analysis to study the enzyme production under the oxygen-limiting conditions of *Aspergillus niger*, which provides a holistic optimization target for industrial application. In this case, it was deduced that the integration of ARTP mutagenesis and multi-omics methods could improve the chances of mining active compounds in a more comprehensive way.

The lasso peptide is post-translationally modified by specific enzymes in which the peptide tails are trapped and locked in a ring (Li et al., 2014). This lasso topology makes the cyclic peptide very stable and difficult to obtain by chemical synthesis (Martin-Gomez and Tulla-Puche, 2018). With the development of in-depth sequencing technology of microbial genomics, there are two primary methods for the exploration of lasso peptide compounds, including screening methods of the McjB homology sequence and centering on the precursor to excavate lasso peptides (Semenova et al., 2005; Maksimov et al., 2012). Hence, multi-omics comparative analysis offers certain advantages over solely gene mining or other sequencing and screening methods. Moreover, it was speculated that iterative ARTP mutagenesis may activate silent gene clusters of secondary substances related to the generation of ribosome biosynthesis. In addition, the putative compounds mycosporines and mycosporine-like amino acids were considered as promising secondary metabolites for UV-photoprotection, and may be widely employed in chemical industries such as cosmetics and sunscreens (Kageyama and Waditee-Sirisattha, 2018). Deoxyloganin generally serves as a precursor of indole alkaloids (Battersby et al., 1970). Fraction F with an unknown compound structure also exhibited obvious bacteriostatic activity, which likely indicates a novel chemical.

Although mutants with strong antibacterial activities were obtained, the compounds extracted with the targeted molecular weights could not be identified due to the limited production. Moreover, the secondary metabolites of actinomycetes may be composed of a series of active components without the main active compounds, which may account for the failure of the separation process (Cai et al., 2002). The host strains were endowed with limited carrying capacity to express the active products, even under artificial or external pressures (Nepal and Wang, 2019). This could be reflected in the low expression of DEGs in the transcriptome profiles, which generally range from a few dozen to a few hundred FPKM. This suggests that even if the cryptic gene cluster could be awakened to express corresponding compounds above the test line, a sufficient threshold may not be achieved to allow for structural identification. Here the higher peak fractions, G and H, had no antibacterial activities, while other fractions with smaller peaks in the liquid chromatogram exhibited bioactivities, indicating that the ARTP mutagenesis activated the silenced gene clusters, however, failed to make induce high expression. Furthermore, several unknown promoters of secondary metabolic gene clusters were supposed to be inactive under laboratory conditions, which may cause short gene expression even if the gene cluster is functional (Myronovskyi and Luzhetskyy, 2016). The cluster activation may presumably disturb the original gene balance, which is non-negligible for the successful gene expression of the comprehensive metabolic pathway (Smanski et al., 2014). Moreover, inappropriate cultural conditions, or an inability to generate environmental signals necessary for streptomycetes in their natural environment, may impact the bioactivity yields (Craney et al., 2012). Future research should focus on improving the expression of these gene clusters or the yields of active compounds. In conclusion, our study provides an efficient method for the detection and identification of targeted bioactive complexes from extracted metabolite crudes of microbial culture mediums.

## Data Availability Statement

The datasets presented in this study can be found in online repositories. The names of the repository/repositories and accession number(s) can be found below: NCBI SRA, accession no: PRJNA679680.

## Author Contributions

TL was mainly responsible for the separation and purification of compounds, omics analysis, and article writing. ZH provided guidance on mutagenesis. XG, WX, and YJ were responsible for sampling and bacterial preliminary screening. JC provided guidance on omics analysis. JZ provided guidance on manuscript modification and overall framework construction. All authors contributed to the article and approved the submitted version.

## Conflict of Interest

The authors declare that the research was conducted in the absence of any commercial or financial relationships that could be construed as a potential conflict of interest.

## Footnotes

1 http://www.ncbi.nlm.nih.gov

2 https://github.com/BenLangmead/bowtie2

3 http://www.biomedsearch.com/nih/RSEM-accurate-transcriptquantification-from/21816040.html

4 http://www.bioconductor.org/packages/2.12/bioc/html/edgeR.html

5 http://www.blats2go.com/b2ghome

6 http://www.genome.jp/kegg

7 http://www.ncbi.nlm.nih.gov/COG/

8 http://mzmine.github.io/
