# Supplementary material for: Multi-omics Comparative Analysis of Streptomyces Mutants Obtained by Iterative Atmosphere and Room-Temperature Plasma Mutagenesis
Source: Front Microbiol. 2021 Jan 28;11:630309. doi: 10.3389/fmicb.2020.630309 (PMC7876522; doi:10.3389/fmicb.2020.630309)
Supplement: Supplementary file 1 [file Data_Sheet_1.docx]

**Supplementary**

**Multi-omics comparative analysis of *Streptomyces* mutants obtained by iterative atmosphere and room-temperature plasma mutagenesis**

Tan Liu^1^, Zhiyong Huang^2^, Xi Gui^1^, Wei Xiang^1^, Yubo Jin^1^, Jun Chen^1^, Jing Zhao^1,3^*

^1^ *College of Ocean and Earth Science of Xiamen University，Xiamen, 361005, China*

^2^ *Tianjin Institute of Industrial Biotechnology, Chinese Academy of Sciences, Tianjin, 300308, China*

^3^ *Fujian Collaborative Innovation Center for Exploitation and Utilization of Marine Biological Resources, Xiamen, 361005, China*

*Corresponding author: Jing Zhao

Address: Xiangan District, Zhoulongquan Building, Xiamen University

Tel.: +86-592-2880811；Fax: +86-592-2880172;

*E-mail address:* [sunnyzhaoj@xmu.edu.cn](mailto:sunnyzhaoj@xmu.edu.cn)

ORCID: 0000-0003-1895-7620

Tan Liu

Tel.: +86-592-2880172; Fax: +86-592-2880172;

E-mail address: [liutan@stu.xmu.edu.cn](mailto:22320161151336@stu.xmu.edu.cn)

Zhiyong Huang

Tel.: 022-84861931; Fax: 022-84861931;

E-mail address: huang_zy@tib.cas.cn

Xi Gui

Tel.: +86-592-2880172; Fax: +86-592-2880172;

E-mail address: xigui@stu.xmu.edu.cn

Wei Xiang

Tel.: +86-592-2880172; Fax: +86-592-2880172;

E-mail address: xiangwei@stu.xmu.edu.cn

Yubo Jin

Tel.: +86-592-2880172; Fax: +86-592-2880172;

E-mail address: 22320201151279@stu.xmu.edu.cn

Jun Chen

Tel.: +86-592-2880172; Fax: +86-592-2880172;

E-mail address: [junchen@xmu.edu.cn](mailto:dxwang@xmu.edu.cn)

Table S1. The number of mutant strains and positive rates obtained by three rounds ARTP mutagenesis

Table S3. The primers of genes with significant differences in functional gene clusters of different mutant stains

Table S4. The result of qRT-PCR

Fig. S1 The correlation coefficien (A) and venn diagram (B) of DEGs between NA, IA and MA in transcriptome profile

Fig. S2 The GO terms (A), COG groups (B) and KEGG pathways (C) of annotated genes

Fig. S3 Linear regression relationship of gene expression levels based on qRT-PCR method

Fig. S4 The fold changes of genes expression obtained based on the qRT-PCR method

Fig. S5 The physicochmical properties of antibacterial active compounds from fermentation supernatant of mutant strain MA

**Table S1. The number of mutant strains and positive rates obtained by three rounds ARTP mutagenesis**

| Iterative round | Total number of mutant strains | The number of antimicrobial activity screening strains | | | | Total number of active strains | Positive mutation rate（%） |
| --- | --- | --- | --- | --- | --- | --- | --- |
|  |  | － | ＋ | ＋＋ | ＋＋＋ |  |  |
| 1 | 234 | 213 | 17 | 3 | 1 | 21 | 8.97 |
| 2 | 259 | 221 | 30 | 5 | 3 | 38 | 14.67 |
| 3 | 320 | 189 | 68 | 60 | 3 | 131 | 40.94 |

Note: “-” meant inactive strains; “+” meant low active strains with bacteriostatic diameter 0 ~ 1.60 cm; “++” meant moderately active strains with bacteriostatic diameters 1.60 ~ 2.20 cm; “+++” meant strains with significantly increased antibacterial activity, bacteriostatic diameters exceeded 2.20 cm

**Table S3. The primers of genes with significant differences in functional gene clusters of different mutant stains**

| Seq ID | Gene name | F-primer | R-primer | bp |
| --- | --- | --- | --- | --- |
| 16S rDNA | 16S rDNA | TAACCCAACATCTCACGACAC | ATACACCGGAAAGCATCAGAG | 95bp |
| AC003_RS23145 | YiaA/B two helix domain protein | GACGTGACCAGGTAGAGAAC | GCCGTCGGCATCTACAA | 80bp |
| AC003_RS13675 | LysM peptidoglycan-binding domain-containing protein | CGATGTCGTCGTTCAGCTC | GCGAGTACAAGGTCGTCAAG | 102bp |
| AC003_RS29175 | SigE family RNA polymerase sigma factor | TACGGTGTGCGCTGATTC | TTGTTCGAGGTTGAGAGGATATG | 125bp |
| AC003_RS20655 | lanthionine-containing peptide SapB | TCAGTTGCAGGTCGTGATG | CCTGCAGTCGATGGAGAC | 115bp |
| AC003_RS04390 | TetR/AcrR family transcriptional regulator | AGGTCACGGACTGGAGTT | GACCAGTCTCAGCGACATC | 116bp |

**Table S4. The result of qRT-PCR**

| Transcriptome differential fold change^1^ | | |
| --- | --- | --- |
| Seq ID | logFC(IA/NA) | logFC(MA/IA) |
| AC003_RS23145 | 4.2 | 4.35 |
| AC003_RS13675 | 0.13 | 3.92 |
| AC003_RS29175 | 2.09 | 3.08 |
| AC003_RS20655 | 2.76 | 2.31 |
| AC003_RS04390 | 0.18 | 0.13 |
| qRT-PCR quantitative result（FC=2^-△△Ct^）^2^ | | |
| Seq ID | logFC(IA/NA) | logFC(MA/IA) |
| AC003_RS23145 | 0.34 | 0.63 |
| AC003_RS13675 | 0.83 | 0.07 |
| AC003_RS29175 | 0.94 | 0.20 |
| AC003_RS20655 | 0.36 | 0.15 |
| AC003_RS04390 | 0.18 | 0.15 |

Note: 1. FC was the logarithm value of gene expression in transcriptome data divided by gene expression in control sample; 2. **FC=2^-△△Ct^** was used to calculate the logarithm value after dividing the gene expression obtained by qRT-PCR between two samples.


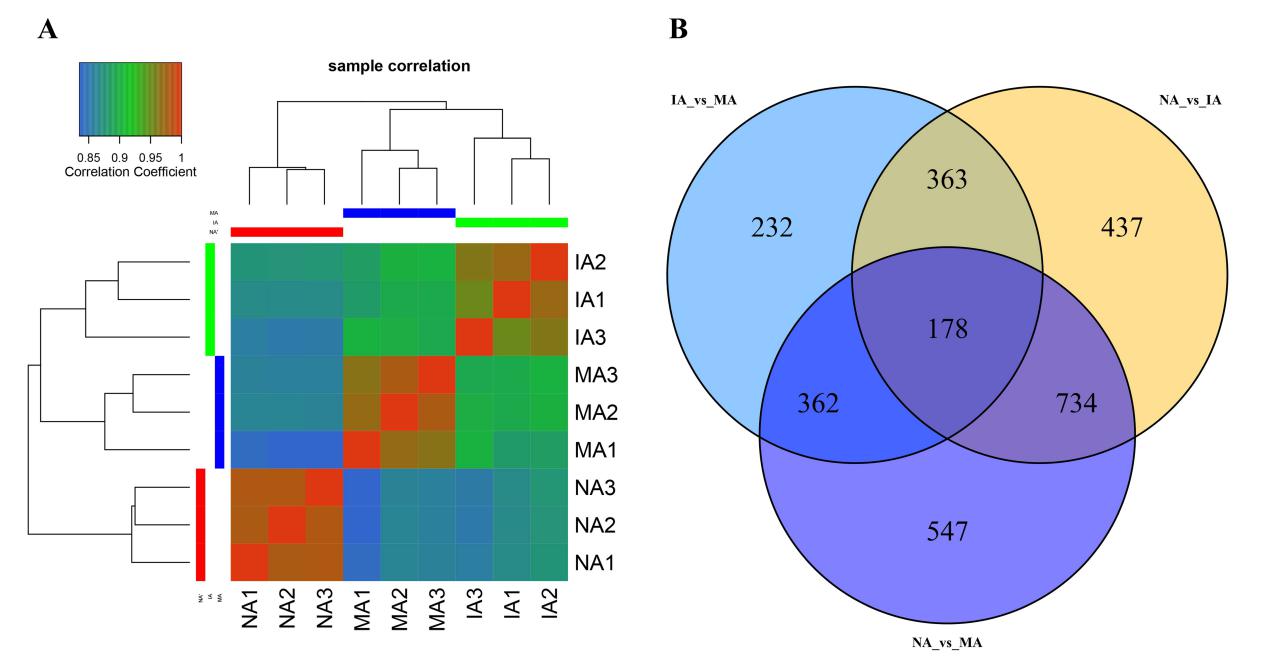


**Fig. S1 The correlation coefficien (A) and venn diagram (B) of DEGs between NA, IA and MA in transcriptome profile**


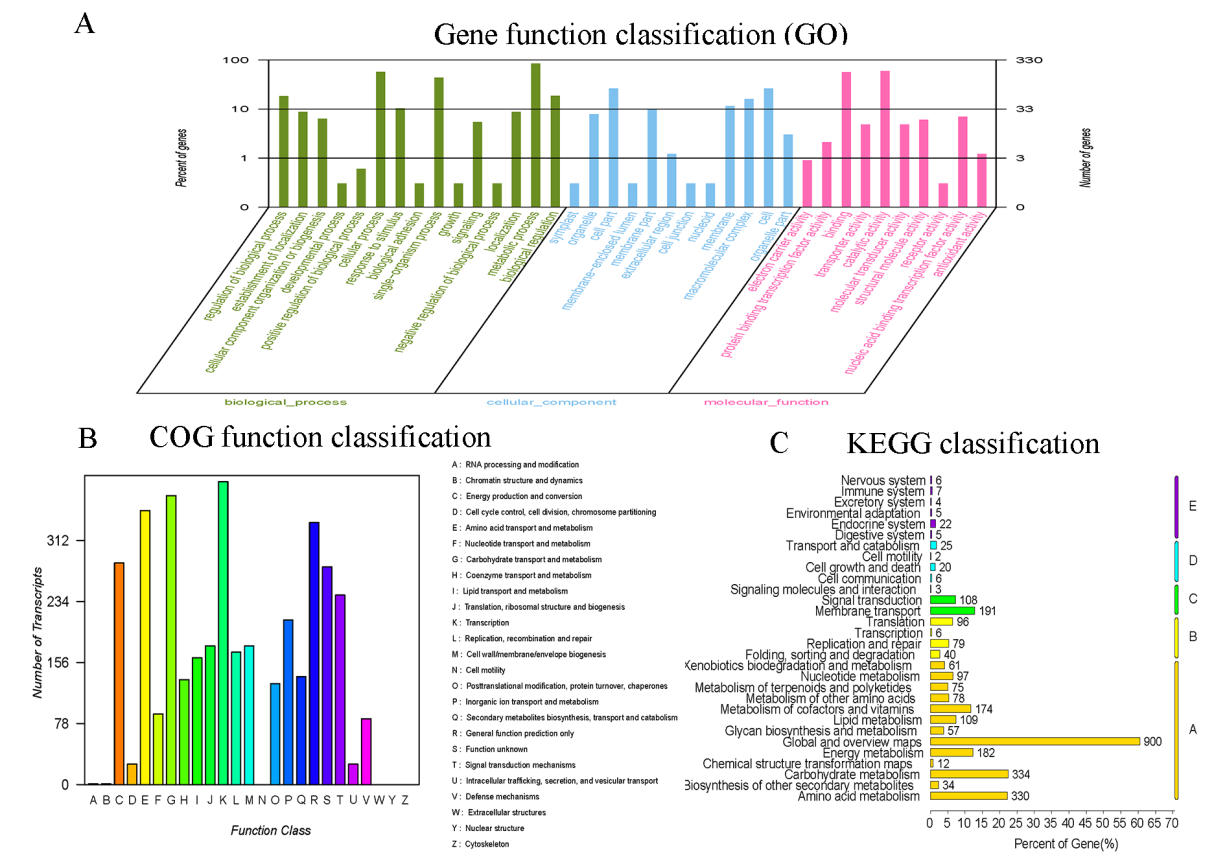


**Fig. S2 The GO terms (A), COG groups (B) and KEGG pathways (C) of annotated genes**


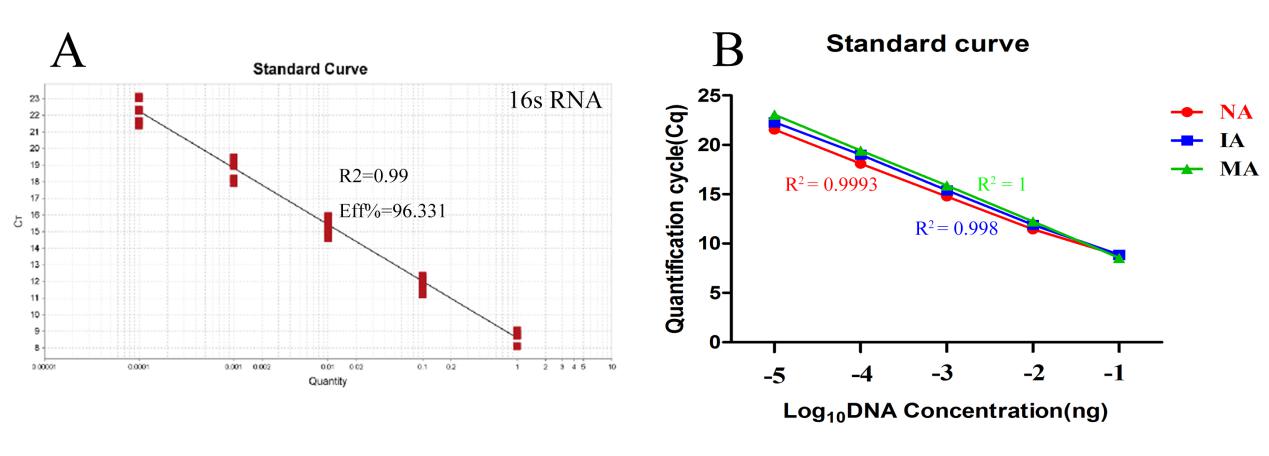


**Fig. S3 Linear regression relationship of gene expression levels based on qRT-PCR method**


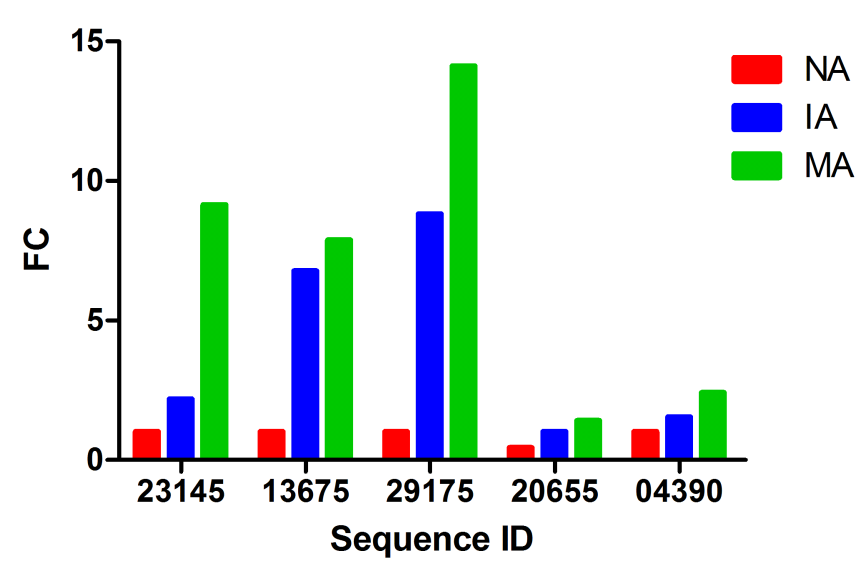


**Fig. S4 The fold changes of genes expression obtained based on the qRT-PCR method**

**Preliminary study on physicochemical properties of antibacterial active compounds**

**Thermal stability analysis**

Mutant strain with increased activity (MA) was inoculated in ISP2 liquid medium and cultivated for 9 days at 28℃. Then 100 μL of supernatant of fermentation broth were incubated at 95℃ water bath for either 1, 2, 4 or 8 hours, separately, or for 4 h at 20℃, 35℃, 50℃, 65℃, 80℃ or 95℃. The antibacterial activity of fermentation broth was measured by Oxford cup method.

**Analysis of protease degradation activity**

Trypsin solution (0.5 μg trypsin was dissolved in 50 mM tris-HCl and 1 mM CaCl_2_, PH 7.6) and chymotrypsin solution (0.5 μg chymotrypsin was dissolved in 100 mM tris-HCl and 10 mM CaCl_2_, PH 8.0)were separately incubated with the supernatant of fermentation broth at 25℃ for 4 h. The antibacterial activity of fermentation broth was also measured by Oxford cup method.


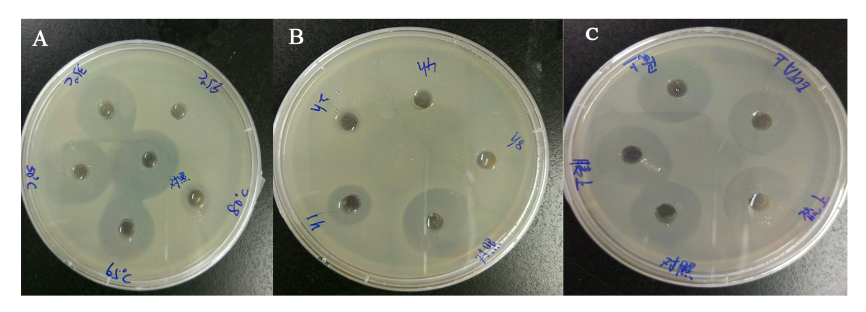


**Fig. S5 The physicochmical properties of antibacterial active compounds from fermentation supernatant of mutant strain MA**

**(A) and (B) Thermal stability analysis; (C) protease degradation activity analysis**
