# Supplementary material for: Multi-omics Comparative Analysis of Streptomyces Mutants Obtained by Iterative Atmosphere and Room-Temperature Plasma Mutagenesis
Source: Front Microbiol. 2021 Jan 28;11:630309. doi: 10.3389/fmicb.2020.630309 (PMC7876522; doi:10.3389/fmicb.2020.630309)
Supplement: Supplementary file 2 [file Data_Sheet_2.docx]

**Table S2. Gene ontology(GO) enrichment analysis of significantly differently expressed genes of different mutant stains**

| GO ID | *P*-value | Total | Up-  regulation | Down-  regulation | Ontology  class | Description |
| --- | --- | --- | --- | --- | --- | --- |
| IA_vs_MA | | | | | | |
| GO:0009059 | 5.86E-04 | 16 | 13 | 3 | biological_process | macromolecule biosynthetic process |
| GO:0005198 | 6.45E-04 | 10 | 10 | 0 | molecular_function | structural molecule activity |
| GO:0042254 | 7.12E-04 | 9 | 9 | 0 | biological_process | ribosome biogenesis |
| GO:0003735 | 7.12E-04 | 9 | 9 | 0 | molecular_function | structural constituent of ribosome |
| GO:0022613 | 7.12E-04 | 9 | 9 | 0 | biological_process | ribonucleoprotein complex biogenesis |
| GO:0006412 | 1.21E-03 | 9 | 9 | 0 | biological_process | translation |
| GO:0044085 | 1.21E-03 | 9 | 9 | 0 | biological_process | cellular component biogenesis |
| GO:0044267 | 2.46E-03 | 10 | 9 | 1 | biological_process | cellular protein metabolic process |
| GO:1990904 | 3.04E-03 | 9 | 9 | 0 | cellular_component | ribonucleoprotein complex |
| GO:0030529 | 3.04E-03 | 9 | 9 | 0 | cellular_component | intracellular ribonucleoprotein complex |
| GO:0071840 | 4.54E-03 | 9 | 9 | 0 | biological_process | cellular component organization or biogenesis |
| GO:0043043 | 4.54E-03 | 9 | 9 | 0 | biological_process | peptide biosynthetic process |
| GO:0034645 | 5.40E-03 | 11 | 10 | 1 | biological_process | cellular macromolecule biosynthetic process |
| GO:0019538 | 5.49E-03 | 13 | 10 | 3 | biological_process | protein metabolic process |
| GO:0006518 | 6.55E-03 | 9 | 9 | 0 | biological_process | peptide metabolic process |
| GO:0043604 | 7.04E-03 | 10 | 10 | 0 | biological_process | amide biosynthetic process |
| GO:0044271 | 1.09E-02 | 14 | 12 | 2 | biological_process | cellular nitrogen compound biosynthetic process |
| GO:0044260 | 1.30E-02 | 19 | 14 | 5 | biological_process | cellular macromolecule metabolic process |
| GO:0044249 | 1.37E-02 | 18 | 14 | 4 | biological_process | cellular biosynthetic process |
| GO:0043170 | 1.55E-02 | 22 | 15 | 7 | biological_process | macromolecule metabolic process |
| GO:0005840 | 1.55E-02 | 7 | 7 | 0 | cellular_component | ribosome |
| GO:0008152 | 1.71E-02 | 51 | 35 | 16 | biological_process | metabolic process |
| GO:0000988 | 1.83E-02 | 4 | 2 | 2 | molecular_function | transcription factor activity, protein binding |
| GO:0016987 | 1.83E-02 | 4 | 2 | 2 | molecular_function | sigma factor activity |
| GO:0000990 | 1.83E-02 | 4 | 2 | 2 | molecular_function | transcription factor activity, core RNA polymerase binding |
| GO:0000996 | 1.83E-02 | 4 | 2 | 2 | molecular_function | core DNA-dependent RNA polymerase binding promoter specificity activity |
| GO:0006352 | 1.83E-02 | 4 | 2 | 2 | biological_process | DNA-templated transcription, initiation |
| GO:0043232 | 2.18E-02 | 7 | 7 | 0 | cellular_component | intracellular non-membrane-bounded organelle |
| GO:0043229 | 2.18E-02 | 7 | 7 | 0 | cellular_component | intracellular organelle |
| GO:0043228 | 2.18E-02 | 7 | 7 | 0 | cellular_component | non-membrane-bounded organelle |
| GO:0043226 | 2.18E-02 | 7 | 7 | 0 | cellular_component | organelle |
| GO:0017000 | 2.84E-02 | 2 | 1 | 1 | biological_process | antibiotic biosynthetic process |
| GO:0016999 | 2.84E-02 | 2 | 1 | 1 | biological_process | antibiotic metabolic process |
| GO:0017144 | 2.84E-02 | 2 | 1 | 1 | biological_process | drug metabolic process |
| GO:0043603 | 2.97E-02 | 10 | 10 | 0 | biological_process | cellular amide metabolic process |
| GO:0009058 | 3.65E-02 | 19 | 15 | 4 | biological_process | biosynthetic process |
| GO:1901576 | 3.86E-02 | 17 | 13 | 4 | biological_process | organic substance biosynthetic process |
| GO:0044444 | 4.09E-02 | 9 | 9 | 0 | cellular_component | cytoplasmic part |
| GO:0019843 | 4.98E-02 | 4 | 4 | 0 | molecular_function | rRNA binding |
| NA_vs_MA | | | | | | |
| GO:0044282 | 5.34E-03 | 4 | 2 | 2 | biological_process | small molecule catabolic process |
| GO:0044712 | 5.34E-03 | 4 | 2 | 2 | biological_process | single-organism catabolic process |
| GO:0000988 | 1.83E-02 | 5 | 3 | 2 | molecular_function | transcription factor activity, protein binding |
| GO:0016987 | 1.83E-02 | 5 | 3 | 2 | molecular_function | sigma factor activity |
| GO:0000990 | 1.83E-02 | 5 | 3 | 2 | molecular_function | transcription factor activity, core RNA polymerase binding |
| GO:0000996 | 1.83E-02 | 5 | 3 | 2 | molecular_function | core DNA-dependent RNA polymerase binding promoter specificity activity |
| GO:0006352 | 1.83E-02 | 5 | 3 | 2 | biological_process | DNA-templated transcription, initiation |
| GO:0015992 | 1.98E-02 | 3 | 1 | 2 | biological_process | proton transport |
| GO:0046395 | 1.98E-02 | 3 | 2 | 1 | biological_process | carboxylic acid catabolic process |
| GO:0016054 | 1.98E-02 | 3 | 2 | 1 | biological_process | organic acid catabolic process |
| GO:0009063 | 1.98E-02 | 3 | 2 | 1 | biological_process | cellular amino acid catabolic process |
| GO:0006818 | 1.98E-02 | 3 | 1 | 2 | biological_process | hydrogen transport |
| GO:1901606 | 1.98E-02 | 3 | 2 | 1 | biological_process | alpha-amino acid catabolic process |
| GO:1901361 | 2.09E-02 | 4 | 2 | 2 | biological_process | organic cyclic compound catabolic process |
| GO:0046700 | 2.09E-02 | 4 | 2 | 2 | biological_process | heterocycle catabolic process |
| GO:0044270 | 2.09E-02 | 4 | 2 | 2 | biological_process | cellular nitrogen compound catabolic process |
| GO:0019439 | 2.09E-02 | 4 | 2 | 2 | biological_process | aromatic compound catabolic process |
| GO:0032774 | 2.89E-02 | 6 | 4 | 2 | biological_process | RNA biosynthetic process |
| GO:0016491 | 4.20E-02 | 18 | 8 | 10 | molecular_function | oxidoreductase activity |
| GO:0016747 | 4.94E-02 | 4 | 2 | 2 | molecular_function | transferase activity, transferring acyl groups other than amino-acyl groups |
| GO:1901565 | 4.94E-02 | 4 | 2 | 2 | biological_process | organonitrogen compound catabolic process |
| GO:0042967 | 4.94E-02 | 4 | 2 | 2 | biological_process | acyl-carrier-protein biosynthetic process |
